# Perceived Risk and Fashion on the Intention to Adopt Wireless Earbuds in the United States Using a Partial Least Squares-Structural Equation Modeling Approach: Empirical Study

**DOI:** 10.2196/56887

**Published:** 2025-06-19

**Authors:** Edward M Lee, Chandrasekar Subramaniam, Sungjune Park

**Affiliations:** 1 Bank of America, Charlotte, NC, United States; 2 Department of Business Information Systems and Operations Management, University of North Carolina Charlotte, 9201 University City Blvd, Charlotte, NC, 28223, United States, 1 7046878622

**Keywords:** wireless earbuds, health risk, wearable comfort, fashionability, intention to buy

## Abstract

**Background:**

The number of studies on the use of smart wearables has increased dramatically in recent years. However, aspects including personal safety and fashion perspectives of wearable devices have not yet been adequately addressed in the literature. There have been debates regarding the potential health risks and fashionability of using wearable devices. Regardless of the actual impact of such devices, these aspects may influence users’ perceptions toward the purchase and use of wearable technology.

**Objective:**

This paper addresses the following research question: How do perceptions of risk and fashion affect the user’s intention to purchase and use wireless earbuds?

**Methods:**

A survey was administered to assess perceptions on health and privacy risks, fashionability, and wearable comfort of wireless earbuds, alongside questions on behavioral intention regarding their purchase and use. All questions were adapted from prior research and measured using a 7-point Likert scale. The final sample of 205 responses was analyzed using the partial least squares method with Smart-PLS software.

**Results:**

Perceived health risk (*P*=.015), perceived fashionability (*P*<.001), and wearable comfort (*P*=.007) had a significant impact on a consumer’s intention to purchase wireless earbuds. Privacy risk did not have a significant impact on intention to purchase. Intention to purchase had a significant impact on intention to use (*P*<.001).

**Conclusions:**

As new types of emerging technology are introduced to the market, technology acceptance models should evolve to better understand consumers’ perceptions toward these new technologies, from both academic and practical points of view.

## Introduction

### Background

Wearable technology is no longer unfamiliar to consumers. According to the International Data Corporation, the worldwide market for wearable devices grew by approximately 20% in 2021, with around 530 million units sold in the third quarter of 2021. Of these units, 339 million were earwear. According to Niknejad et al [1], the publication of studies relating to smart wearables has also increased dramatically in recent years. Less than 50 studies on wearable devices were published in 2013, rising to 404 in 2022 [2]. These studies covered topics ranging from technical issues, user behavior, design, security, and privacy to the issue of social acceptability. However, personal safety perspectives of wearable devices have not been adequately addressed in the literature to date.

Debates continue regarding the health risks of wearable devices. For instance, some researchers have raised concerns about wireless earbuds due to their high levels of radio-frequency radiation, while others have contested this risk [3]. Other researchers have suggested that powerful batteries close to the human body for extended periods could cause leukemia, but this has also been contested [4]. Regardless of the actual health risks associated with wearable devices, these debates can influence and shape users’ perceptions toward wearable technologies [5]. It is important to understand how perceived health risks impact consumers’ perceptions of wearable devices.

A unique feature of wearable devices is that they are not only considered technical devices but also as fashion items. Fashion-related factors affect consumers in their evaluation of wearable technologies [6]. Wireless earbuds are available in different colors, and smartwatches have options such as changeable straps for consumer design preference. The roles of perceived risk and fashion in a consumer’s choice to purchase and use wearable devices have not been studied in the literature.

This paper addresses the following research question: *Do perceptions of technology, risk, and fashionability affect the user’s intention to purchase wireless earbuds?* This study will fill the gap in the literature of a systematic understanding of the impact of perceived risk and fashionability on wearable device purchase and use. The results of our study will also help the development of safer wearable devices. The remainder of the paper is organized as follows. The next section reviews the relevant literature and presents the research gaps. The section "Research Model and Hypotheses" presents our research model and hypotheses, while "Method" describes our research method and results. The research findings are then discussed, and we conclude by presenting the study’s limitations and future research opportunities.

### Literature Review

The underlying theory for our study is the Unified Theory of the Adoption and Use of Technology 2 (UTAUT 2). The UTAUT 2 theory is complemented by the literature on the perceived risks and fashionability of wearable devices to build our model. This section synthesizes the current literature in the above 3 research streams.

Wearable devices or technologies are mobile technologies that are integrated into clothing and accessories, incorporating wireless connectivity for access, interaction, and the exchange of information anytime and anywhere [7]. Wearable technology is used in various industries. For example, end consumers wear wireless earbuds to listen to music or answer phone calls, while the health care industry uses wearable trackers for patient and disease management. This study will focus on the consumers’ perspectives and their behavioral intention to purchase and use wireless earbuds, a type of wearable device.

The underlying theory, UTAUT 2, was developed to understand technology acceptance in a consumer use context [8]. UTAUT 2 extends the original UTAUT model and incorporates three additional constructs: hedonic motivation, price value, and habit. Hedonic motivation is “the fun or pleasure derived from using a technology” and plays a critical role in determining technology acceptance and use [9]. Price value is “the consumers’ cognitive tradeoff between the perceived benefits of the applications and the monetary cost for using them” [10]. Habit is “the extent to which people tend to perform behaviors automatically because of learning” [11].

Literature on wearable device use has covered themes including user behavior and aspects of the wearables themselves, such as technological features, design, security and privacy issues, and social acceptability [1]. For example, Grym et al [12] studied the feasibility of smart wristband wearable use among first-time pregnant women and found that the device usage was similar during the second and third trimesters but decreased during the postpartum period. The authors identified various reasons for the decreased usage, including problems with charging the devices, a perception of the devices as uncomfortable, and fear of scratching their babies with the devices.

Previous research on user behavior has examined users’ willingness relating to different aspects, such as their intention to purchase, adopt, and use technologies, alongside their actual use and user experiences [1]. For example, Li et al [13] found that among adults older than 60 years, perceived usefulness, compatibility, facilitating conditions, and self-reported health status positively affected their intention to use smart wearable technologies. Nunes and Arruda Filho [14] examined users’ Google Glass adoption and identified three categories of users: socially satisfied, socially constrained, and early adopters. The early adopters were subcategorized as enthusiasts and visionaries. Dehghani and Kim [15] proposed three key factors, such as screen size, uniqueness, and design, that affected participants’ current and potential future purchase intention of smartwatches. From these studies, it is clear that to study the purchase intention of wireless earbuds, we must complement the foundation of UTAUT 2 with additional variables relevant to the use context of wearables.

The health risks associated with wearable devices are highly debated in the literature. One researcher raised concerns about the placement of wireless earbuds in the ear canal, which exposes tissues in the head to relatively high levels of radio-frequency radiation, eventually increasing the chance of cancer [3]. However, another researcher, who has studied the effects of wireless radiation on human health, wrote that there is no health risk from wearable devices and that the arguments about adverse health effects of wearables have no credibility [3]. Researchers have also raised concerns about having powerful batteries close to the human body for an extended period, as being too close to power lines over long times may cause leukemia [4]. In 2015, a group of 250 scientists filed a petition to the World Health Organization (WHO) and United Nations (UN) regarding the health risks of electromagnetic field radio waves generated by wireless devices [3]. However, Heid points out that the WHO and other public health organizations have not found “any clear evidence for health hazards at exposure levels below international limits” [16].

Regardless of the actual health risks of wearable devices, these debates on adverse health effects could influence and shape users’ perceptions of the technology [5]. Attitudes are formed by an individual’s belief regarding the consequences of using a particular technology, whether those consequences are perceived or real [5]. The information available about a particular technology affects the individual’s perceptions and impacts their intention to use that technology [5]. Thus, perceptions regarding health risks can affect consumer behavior, regardless of the actual level of health risk [17].

Due to the COVID-19 pandemic, consumers may be more sensitive to the health risks than before, regardless of the actual health risks of products or technology. For instance, a rumor spread on social media that the pandemic was caused by 5th generation wireless network technology [18]; this was refuted by the United States Federal Emergency Management Agency [18]. As consumers become more sensitive to health risks, the perception of these risks must be studied in the wearable technology. This is an aspect lacking in the literature on wearable technology [1].

The Bluetooth technology, used to power almost all wireless earbuds, expands the attack surface and is subject to security and privacy risks [19]. The privacy attack types include passive eavesdropping and active eavesdropping. When wireless earbuds are used for activities beyond listening to music, such as controlling devices using voice commands, they are vulnerable to compromising the listener’s security and privacy. Researchers have shown that perceived privacy risks have a negative impact on the value perception of wearable devices and on the intention to purchase and use these devices [20].

Perceived fashionability, the perception of the design component of a product, has been known to affect consumer purchase and use of a product [21]. Wearable accessories can strongly impact the user’s physical appearance [6]. Therefore, how consumers view the design of wearable technology devices can impact their intention of using the device. When Apple announced their first wireless headphones called AirPods, people disliked the aesthetic and shared jokes on social media, comparing their design to a toothbrush head [22]. Meanwhile, wearable comfort refers to the “consumers’ overall subjective assessment of the physical feeling from wearing” a device [6]. Comfortable wearable devices increase usage enjoyment. This is related to users’ expectations of functionalities for certain apparel, such as raincoats [6].

Despite the jokes circulating on the Internet regarding the design of Apple’s AirPods, competitors such as smartphone manufacturers have launched their own wireless earbuds. Wireless earbuds are now more sophisticated, with additional features to control other devices, and are likely to increase in popularity among consumers. Therefore, studying consumers’ fashion perceptions of wireless earbuds would provide additional insights into their purchase intention.

### Research Model and Hypotheses

**Figure 1.**
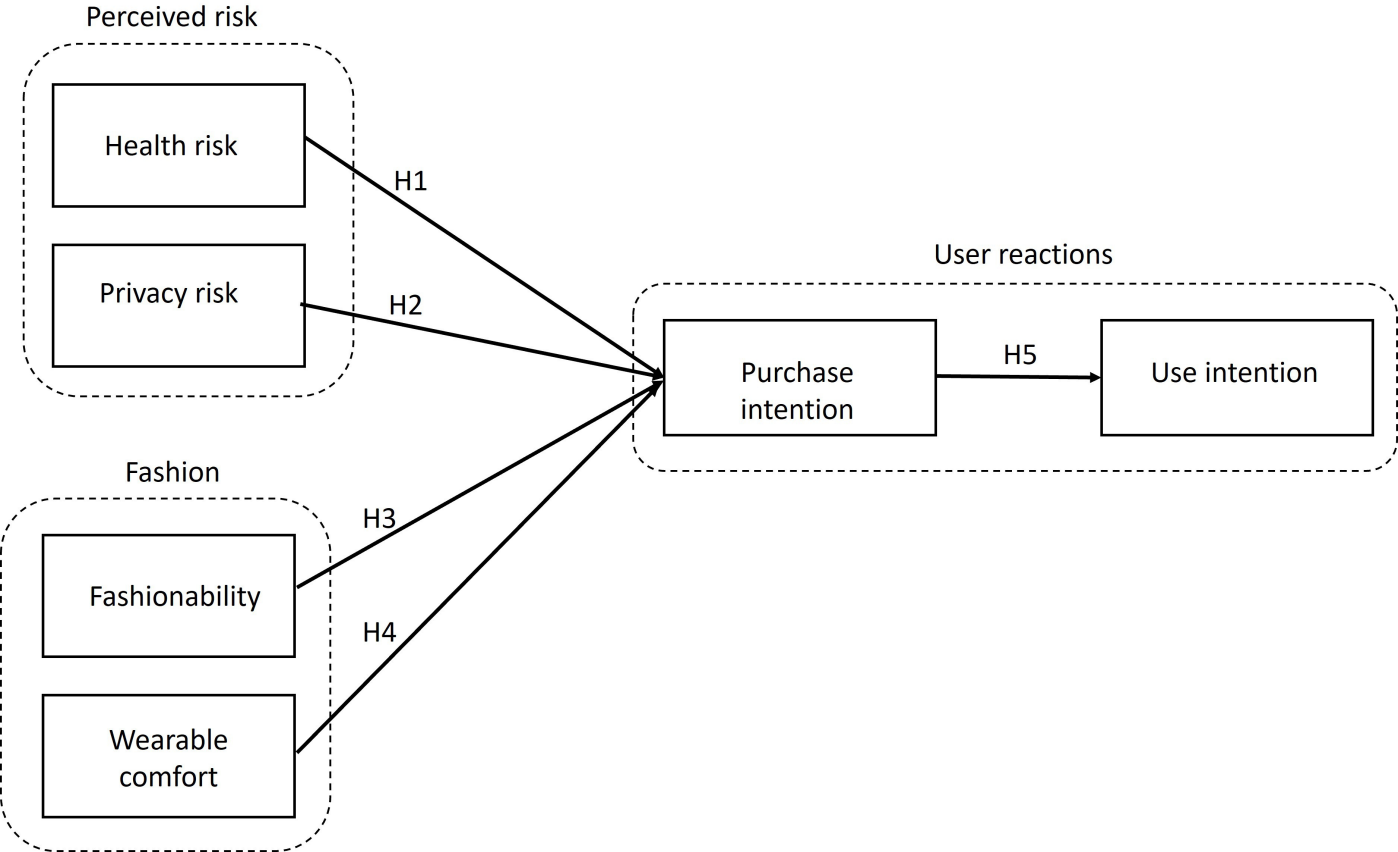
Conceptual model.

In this section, we present our research model on consumers’ intention to purchase and use wireless earbuds, incorporating antecendents from UTAUT 2, perceived risk and perceived fashion. The conceptual model is shown in Figure 1. Below we present our hypotheses.

Despite ongoing debates on the health risks of wearable devices, there is no consensus regarding the actual health effects of using wireless earbuds. While some researchers have expressed concerns about the extended use of wireless technology electronics and rechargeable batteries, others claim a lack of evidence to support these health risks, positing wearable devices as safe for use [3,4,16]. Regardless, such debates on health risks potentially influence consumer perceptions of wearable technology. Consumers are more likely to purchase wireless earbuds if they believe them to be safe. In contrast, consumers are less likely to purchase wireless earbuds if they think long-term use could be harmful to their health due to exposure to wireless radiation. Hence, our first hypothesis is as follows:

#### H1: Perceived Health Risk Has a Negative Influence on the Behavioral Intention to Purchase Wireless Earbuds

Perceived privacy risk is another factor that could influence consumers’ attitudes towards wearable technology [23]. Although wireless earbuds generally only play music, they are connected to a main device, such as a smartphone, through Bluetooth wireless technology. This means third parties might be able to gain access to a smartphone by hacking connected wireless earbuds [19,24]. Wireless earbuds may also allow eavesdropping on sensitive conversations of a consumer and compromise their privacy [19]. Regardless of the actual possibility of such hacking, a consumer’s sensitivity regarding privacy protection could affect how they view wearable devices. If a consumer is concerned with personal information being collected by companies or being exposed to hackers, they may prefer to use wired headsets rather than wireless earbuds. Therefore, we hypothesize that:

#### H2: Perceived Privacy Risk Has a Negative Influence on the Behavioral Intention to Purchase Wireless Earbuds

Wearable technologies are not only evaluated based on their functional quality but also on their perceived fashionability and wearable comfort. Herz and Rauschnabel [6] identified three broad categories of fashion-related factors that explain why people chose particular apparel: perceived fashionability, wearable comfort, and functional quality.

Perceived fashionability is the perception of the design component of a product and has been known to impact consumer behavior [21]. Consumers may choose wearable technology devices based on how they fit with their outfits, and the ownership of wearable accessories can strongly impact one’s physical appearance [6]. Therefore, the design and color of wearable devices can impact a consumer’s intention to use them. For example, the design of Apple’s AirPods was initially criticized on social media, and this could have negatively affected their sales. The study hypothesizes that:

#### H3: Perceived Fashionability Has a Positive Influence on the Behavioral Intention to Purchase Wireless Earbuds

Wearable comfort is the overall subjective assessment of the physical feeling from wearing a device [6]. Since wireless earbuds are typically worn for extended periods, comfort is a critical factor in their evaluation. Consumers may be less likely to use wearable technology devices that are uncomfortable or hinder their daily activities. For example, a consumer would not wear wireless earbuds if they made their ears uncomfortable during jogging or exercising. Therefore, the study hypothesizes that:

#### H4: Wearable Comfort Has a Positive Influence on the Behavioral Intention to Purchase Wireless Earbuds

According to Ajzen [22] , intentions are presumed to be an indicator of to what extent people are willing to perform a certain behavior. Consumers who intend to adopt wireless earbuds will consider purchasing them. Consumers who already own wireless earbuds may consider repurchasing more for different purposes. For example, a consumer may consider ordering a second pair of wireless earbuds so that one set can be used at work and the other can be used while exercising. There could also be cases of consumers who already own wireless earbuds who consider buying next-generation devices as an upgrade. Following the intention to purchase, consumers will consider how frequently they would use wireless earbuds. Therefore, it is hypothesized that behavioral intention to purchase drives use intentions.

#### H5*:* Behavioral Intention to Purchase Wireless Earbuds Has a Significant Influence on Behavioral Intention to Use Wireless Earbuds

In summary, the main model includes perceived health risk and privacy risk to understand the impact of perceived risk on consumers’ attitudes toward wireless earbuds. In addition, fashionability and wearable comfort from Herz and Rauschnabel [6] were added to incorporate consumers’ perceptions from an aesthetic view. Figure 1 is the conceptual model of this study. The definitions of the model constructs are provided in Table 1.

**Table 1.** Definition of perception constructs.

Construct	Study	Definition
Health risk	Jacoby and Kaplan [25]	“The risk to the buyer’s or other’s safety in using products.”
Privacy risk	Featherman and Pavlou [26]	“Potential loss of control over personal information, such as when information about you is used without your knowledge or permission.”
Fashionability	Homburg et al [21]	“The perception of the design component.”
Wearable comfort	Herz and Rauschnabel [6]	“Consumers’ overall subjective assessment of the physical feeling from wearing.”

In addition to the main variables for the constructs, three control variables were included in this study: age, experience, and awareness of health risks. Age was controlled as there is a higher chance of younger consumers having better knowledge of wearable technology, impacting their reactions toward the technology. Consumers’ reactions toward wireless earbuds would most likely change according to whether they have tried or own earbuds; thus, experience was controlled for. Reactions toward wireless earbuds could be different between consumers who are aware or not aware of potential health risks; thus, awareness of wireless earbuds’ potential health risks was also controlled.

## Methods

### Instrument Development

Each scale used was adapted from previous research. The survey items are included in the Multimedia Appendix 1. The scales for perceived health risk, perceived privacy risk, perceived fashionability, wearable comfort, and behavioral intention to purchase are adapted from Herz and Rauschnabel [6]. Each survey item was measured using a 7-point Likert scale ranging from 1 (“strongly disagree”) to 7 (“strongly agree”). Age was measured in years and experience was coded using a 0 or 1 dummy variable, for which 0 represents “does not own wireless earbuds” and 1 represents “owns wireless earbuds”. Awareness of health risks was coded using a 0 or 1 dummy variable, for which 0 represents “not aware” and 1 represents “aware” of the potential health risks of wireless earbuds. For additional insights, optional questions included the duration of ownership and feedback relating to the purchase and use of wireless earbuds. Duration of ownership was measured in months. Except for duration of ownership and feedback, participants were required to answer every question to prevent missing values.

### Data Collection

The survey included a short description of wireless earbuds and their main functionalities for participants who are not familiar with the devices. Based on the response to the control question regarding experience (whether or not the participant owns wireless earbuds), participants were assigned different question sets; these were mostly identical but used slightly different terms applicable to the participant’s situation. The quality of the survey questions was reviewed and improved, based on responses to a pilot study conducted with 23 participants. For example, in the initial survey, there was confusion about the meaning of “re-purchasing wireless earbuds” for those who had already owned a pair; thus, a brief description was added (“additional pair for different occasions or upgrading to next model”).

The finalized survey was distributed to panelists through Qualtrics, an American experience management company. To gather a diverse and informative sample, we used a purposive sampling strategy. Specifically, 2 distinct groups were targeted, consumers who currently own wireless earbuds and consumers who do not. This deliberate selection allowed the collection of data from both segments of the market to gain a comprehensive understanding of consumer intentions. Participants were all located in the United States and were 18 years old or above. A total of 304 participants accessed the online survey, and 276 of them completed it; however, 71 completed surveys were removed due to incomplete answers. Thus, a total of 205 completed survey datasets were analyzed. The sample size of 205 meets the suggested minimum threshold of “ten times the largest number of structural paths directed at a particular latent construct in the structural model” [24].

### Measurement and Structural Analysis

Partial least squares (PLS) analysis was used to test the model, as PLS is capable of testing the effects of several interaction terms [27]. Using Smart-PLS software (version 3.3.3), the measurement model and structural model were examined. The measurement model, also called the outer model, describes the relationships between the constructs and indicator variables [28]. The structural model, also called the inner model, represents the constructs and describes the relationships between them [28].

The reliability and validity of the measurement model were evaluated by assessing internal consistency reliability, indicator reliability, convergent validity, and discriminant validity . Cronbach alpha, a traditional criterion for internal consistency, provides an estimate of reliability based on the intercorrelations of the observed indicator variables. For reliability to be acceptable, Cronbach alpha for internal consistency reliability should be higher than 0.7. Indicator reliability is determined by the size of the outer loading. For indicator reliability, the standardized indicator loadings should be 0.7 or higher.

Convergent validity is the degree to which a measure correlates positively with alternative measures of the same construct. The average variance extracted (AVE), a common measure to establish convergent validity on the construct level, is defined as “the grand mean value of the squared loadings of the indicators associated with the construct.” For convergent validity, AVE should be 0.50 or higher, meaning the construct explains more than half of the variance of its indicators on average. Discriminant validity is the degree to which a construct is truly distinct from other constructs by empirical standards. There are two measures of discriminant validity: cross-loadings and the Fornell–Larcker criterion. Cross-loadings describe the correlation between an indicator and other constructs in the model; generally, an indicator’s outer loading on its associated construct should be greater than its correlation with any other construct. Meanwhile, the Fornell–Larcker criterion compares the square root of each construct’s AVE value with its correlations with other constructs. The square root of each construct’s AVE should be greater than its highest correlation with any other construct in the model.

For evaluating the structural model, the coefficient of determination (*R*^2^ value) and the level and significance of the path coefficients are the primary criteria [24]. *R*^2^ is a “measure of the model’s predictive power and is calculated as the squared correlation between a specific endogenous construct’s actual and predicted values”. For endogenous latent variables, *R*^2^ values of 0.75, 0.50, or 0.25 can be respectively described as substantial, moderate, or weak. The level and significance of the path coefficients are assessed through bootstrapping. In this process, subsamples are randomly drawn from the original sample with replacement [28]. This helps determine whether each relationship in the model is statistically significant and whether they support the hypotheses [24]. For this study, 300 subsamples were set for bootstrapping.

In examining the measurement and structural models, survey items with low loadings were removed in order to improve the constructs’ reliability and validity. Removed survey items are indicated in the Multimedia Appendix 1. The PLS algorithm in Smart-PLS provides the reports of construct reliability and validity, discriminant validity, collinearity statistics (variance inflation factor), and *R*^2^. Bootstrapping provides the specific indirect effects report for mediation effects and the path coefficients report for path significance.

### Ethical Considerations

This study was approved by the Office of Research Protections and Integrity, University of North Carolina at Charlotte (review number 21-0223). The study was approved with the statement “Exemption Category: 2. Survey, interview, public observation”.

## Results

### Demographic Statistics

The survey included questions about age, ownership of wireless earbuds, and awareness of health risks. The youngest respondent was 18 years old while the oldest was 89 years. The average age of the respondents was 43 (SD 14.29) years, with the majority falling in the age range of 30-49 years. Among those who owned wireless earbuds, ownership duration ranged from 1 month to 8 years and 4 months, with an average of a year and 4 months. Out of the 205 respondents, 63.4% (130/205) owned wireless earbuds, and 56.6% (116/205) were aware of the potential health risks associated with them.

### Measurement and Structural Model Results

The construct reliability and validity reports provided each construct’s Cronbach alpha and AVE. Cronbach α for every construct exceeded 0.80; thus, all constructs can be said to be reliable. Every construct exhibited an AVE higher than 0.50, suggesting convergent validity. Table 2 presents Cronbach α and AVE values for each construct. Discriminant validity is also presented in the form of the Fornell-Larcker criterion in Table 2. The diagonal cells show the square root of AVE of each construct. No constructs have exceptionally high correlations. This means that the constructs were truly distinct from each other by empirical standards. The correlation between perceived fashionability and wearable comfort is higher than between other variables. However, according to the collinearity statistics report, every variance inflation factor value was below 5.; Thus, we conclude that the constructs are acceptable for further analysis.

**Table 2.** Discriminant validity using the Fornell–Larcker criterion.

Model Construct	Purchase intention	Use intention	Fashionability	Health risk	Privacy risk	Wearable comfort
Purchase intention	0.901					
Use intention	0.840	0.924				
Fashionability	0.668	0.658	0.920			
Health risk	–0.422	–0.442	–0.319	0.748		
Privacy risk	–0.137	–0.175	–0.110	0.534	0.774	
Wearable comfort	0.642	0.626	0.676	–0.406	–0.152	0.879
Cronbach α	0.884	0.914	0.820	0.806	0.845	0.853
Average Variance Extracted (AVE)	0.812	0.853	0.847	0.560	0.599	0.773
Composite reliability	0.928	0.946	0.917	0.834	0.852	0.911

**Figure 2.**
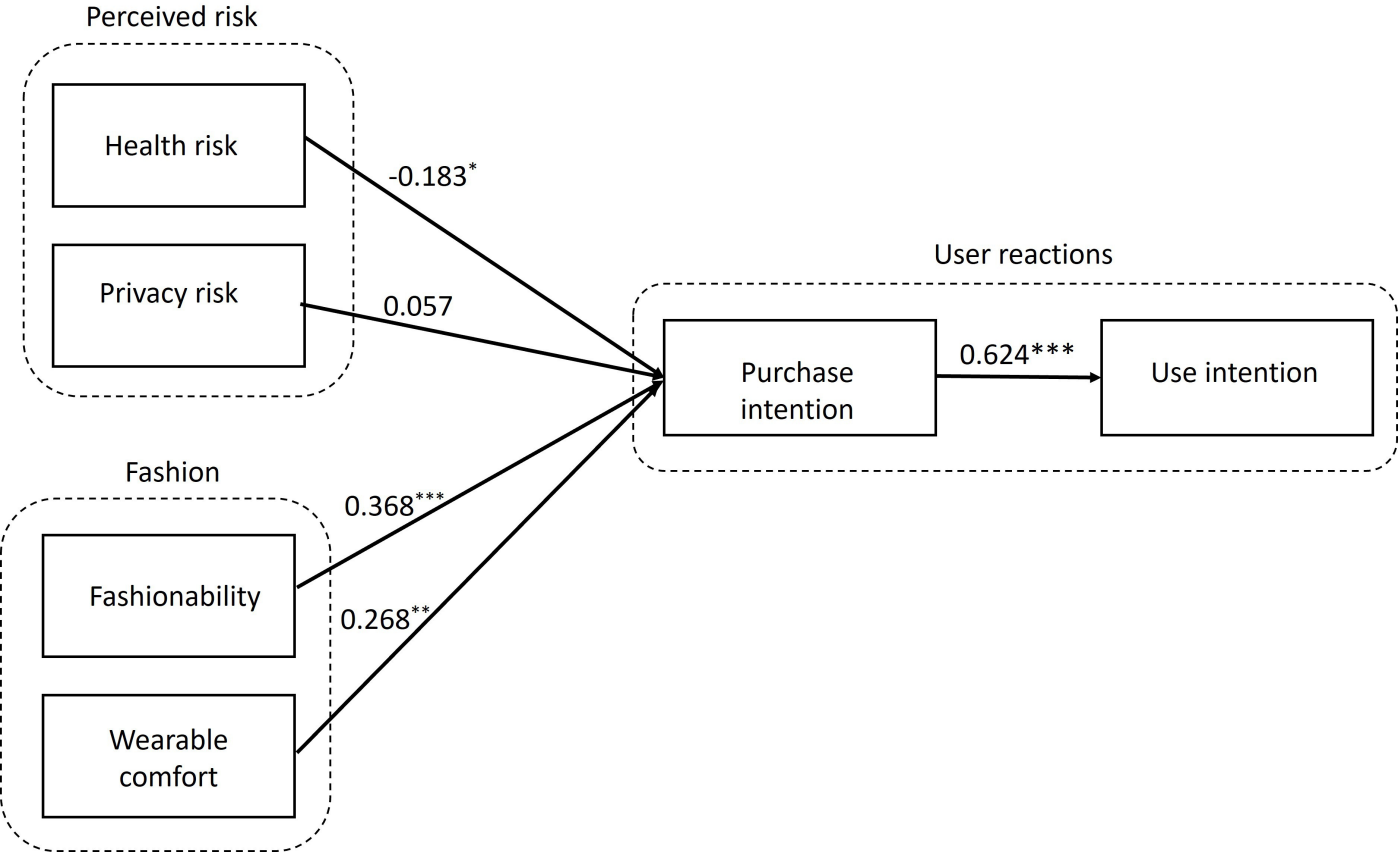
Final model results – path coefficients and statistical significance. *P<.05, **P<.01, ***P<.001

The PLS result shows that *R*^2^ and adjusted *R*^2^ values for purchase intention were 0.560 and 0.544, respectively, and R^2^ and adjusted R^2^ of behavioral intention to use were 0.757 and 0.747, respectively. Therefore, the model’s predictive power is substantial. The specific indirect effects report provides the significance of the mediator. As shown in Table 3, this was statistically significant (*P*<.05), suggesting that behavioral intention to purchase mediates the relationship of perceived health risk, perceived fashionability, and wearable comfort with behavioral intention to use. However, the mediation of the relationship between perceived privacy risk and behavioral intention to use by behavioral intention to purchase was not found to be statistically significant. The path coefficients report provides the significance of each path in the model. As shown in Table 3, the *P* values for the indirect effects of perceived fashionability, perceived health risk, and wearable comfort on behavioral intention to use, mediated through behavioral intention to purchase, were all less than .05. The relationship between behavioral intention to purchase and behavioral intention to use was also statistically significant (Table 4). Hypotheses H1, H3, H4, and H5 are therefore supported by the data. However, the *P* value for the indirect effect of perceived privacy risk was greater than .05. Therefore, H2 is not supported. The overall results are illustrated in Figure 2.

**Table 3.** Specific indirect effects.

Path	Original sample	Sample mean (SD)		*P* value
Privacy Risk → Purchase Intention → Use Intention	0.035	0.023 (0.042)		.40
Health Risk → Purchase Intention → Use Intention	−0.114	−0.108 (0.048)		.02
Fashionability → Purchase Intention → Use Intention	0.230	0.223 (0.061)		<.001
Wearable Comfort → Purchase Intention → Use Intention	0.167	0.167 (0.067)		.01

**Table 4.** Path coefficients.

Paths	Original sample	Sample mean (SD)		*P* value
Purchase Intention → Use Intention	0.624	0.621 (0.076)		<.001
Fashionability → Purchase Intention	0.368	0.359 (0.084)		<.001
Fashionability → Use Intention	0.124	0.122 (0.061)		.04
Health Risk → Purchase Intention	–0.183	–0.174 (0.075)		.02
Health Risk → Use Intention	–0.049	–0.053 (0.047)		.30
Privacy Risk → Purchase Intention	0.057	0.040 (0.069)		.41
Privacy Risk → Use Intention	–0.022	–0.020 (0.052)		.68
Wearable Comfort → Purchase Intention	0.268	0.269 (0.098)		.007
Wearable Comfort → Use Intention	0.059	0.061 (0.054)		.278

Table 4 presents the direct path coefficients, further highlighting the relationships among variables. Perceived fashionability had a direct effect on use intention, indicating a complementary partial mediation, whereas the direct effects of perceived health risk and wearable comfort on use intention were not statistically significant, indicating a full mediation.

## Discussion

### Principal Findings

Based on the results of this study, perceived health risk, perceived fashionability, and wearable comfort have a significant impact on consumers’ decisions in terms of purchasing wireless earbuds. The significance of perceived health risk fills a gap in the literature on wearable technology, which has not been previously adequately studied. Perceived health risks had a significant negative impact on the intention to purchase wireless earbuds; however, they did not have a significant impact on the intention to use the devices. This could mean that even before considering using wireless earbuds, consumers may not purchase them at all due to the potential health risks. However, once consumers had purchased wireless earbuds, the perceived health risks did not seem to affect their subsequent usage. Poor sales impact business; thus, it is important for developers to make sure that their technology and products are fully tested and safe to use before introducing them to the market. In addition, producers should help consumers understand that their products are safe to use. Perceived privacy risk did not have a significant impact on either the intention to purchase or use wireless earbuds. This could mean that consumers do not consider potential privacy risks when they purchase and use wireless earbuds, or they may not be aware of the risk. In the qualitative responses to the survey, none of the respondents mentioned privacy risks among their concerns. In future research, adding a survey question asking if the respondent is aware of any privacy risks from using wearable devices would provide more insight.

The significance of perceived fashionability and wearable comfort reaffirms that consumers view wearable devices as not only technical devices but also as fashion items. Perceived fashionability has a significant impact on the intention to both purchase and use wireless earbuds. This means consumers may not purchase and use a wearable device if they think the product is ugly. Developers should make sure to produce wearable devices that are not only highly technical but also fashionable. Furthermore, wearable devices must not only be fashionable but also comfortable to wear.

Comparing the path coefficients and effect size between the three significant variables (perceived health risk, perceived fashionability, and wearable comfort) provides insight into how impactful each variable is. Wearable comfort had the highest path coefficient (0.344), with perceived fashionability second (0.285) and perceived health risk the lowest (−0.202). The effect size, also known as f-squared, indicates the relevance of variables in explaining selected endogenous constructs [28]. A higher f-squared value means larger effects of the specific variable on endogenous constructs. As with the comparison of path coefficients, wearable comfort exhibited the highest effect size (0.052), with perceived fashionability second (0.041) and perceived health risk the lowest (0.039). The results of the path coefficients and effect sizes imply that wearable comfort has the most impact on the consumer’s intention to purchase wireless earbuds. In other words, consumers consider the fashion of wireless earbuds more than the wireless earbuds’ potential health risks.

### Conclusion

Certain limitations of this study provide opportunities for future research. First, the sample group for the survey was recruited by Qualtrics, which hired respondents through their panel program. Respondents were paid to complete the survey. Studying a larger and truly randomized sample group may help generalize the results. In addition, with a larger sample group, it would be possible to compare results between those who already own and those who do not own a wearable device. The results could vary depending on the type of wearable device, the type of consumer, and how or why the device was purchased. The impact of perceptions could be different when intending to purchase or use wireless earbuds compared to other devices like smartwatches. Adding technology type to the model in future research with a larger sample group could provide a more generalizable result. Different types of consumers will have different perceptions and needs. For example, some survey respondents did not see the need to purchase and use wireless earbuds due to hearing conditions (hearing issues or hearing aids). Patients who need to track their health conditions may have different motivations for using wearable devices. Motivation for purchasing a wearable device would also be different if the buyer and the actual user were different people. For example, perceptions toward wearable devices could be different between parents who are purchasing the device for their children and the children themselves as the actual users of the device. Consumers with different education levels, salaries, or jobs may also have different perceptions toward wearable devices.

The availability of alternatives could be another factor that affects the acceptance of wearable devices. For example, wired headsets are an alternate option to wireless earbuds for listening to music. However, no alternative provides the same functionality that smart glasses offer. A number of respondents also mentioned that they are opposed to using wireless earbuds because they think they could be a distraction or block the user’s surroundings. These concerns could be considered new types of perceived risk. Conducting qualitative as well as quantitative research in the future could provide more insights and ideas for wearable technologies. As technology advances, new types of emerging technology will be introduced to the market. Technology acceptance models and studies must also evolve to understand consumers’ perceptions toward these new technologies from both academic and practical points of view.

## Supplementary material

10.2196/56887 Multimedia Appendix 1 Survey Items.

## Abbreviations

AVE: average variance extracted
PLS: partial least squares
UN: United Nations
UTAUT 2: Unified Theory of the Adoption and Use of Technology 2
WHO: World Health Organization

## References

[1] Niknejad N, Ismail WB, Mardani A (2020). A comprehensive overview of smart wearables: the state of the art literature, recent advances, and future challenges. Eng Appl Artif Intell.

[2] Zhang N, Peng Y, Guo Q (2024). Visual analysis of research trends and hotspots in wearable electronic devices in the medical field: a bibliometric study. Digit Health.

[3] Waugh R (2019). Wireless headphones like apple airpods ‘could pose cancer risk’, scientists warn. Yahoo News.

[4] Bilton N (2015). The health concerns in wearable tech. The New York Times.

[5] Ajzen I, Fishbein M (1980). Understanding Attitudes and Predicting Social Behavior.

[6] Herz M, Rauschnabel PA (2019). Understanding the diffusion of virtual reality glasses: the role of media, fashion and technology. Technol Forecast Soc Change.

[7] Bower M, Sturman D (2015). What are the educational affordances of wearable technologies?. Comput Educ.

[8] Venkatesh V, Thong JY, Xu X (2012). Consumer acceptance and use of information technology: extending the unified theory of acceptance and use of technology. MIS Q.

[9] Brown SA, Venkatesh V (2005). Model of adoption of technology in households: a baseline model test and extension incorporating household life cycle. MIS Q.

[10] Dodds WB, Monroe KB, Grewal D (1991). Effects of price, brand, and store information on buyers’ product evaluations. J Mark Res.

[11] Limayem M, Hirt SG, Cheung CMK (2007). How habit limits the predictive power of intention: the case of information systems continuance. MIS Q.

[12] Grym K, Niela-Vilén H, Ekholm E (2019). Feasibility of smart wristbands for continuous monitoring during pregnancy and one month after birth. BMC Pregnancy Childbirth.

[13] Li J, Ma Q, Chan AH (2019). Health monitoring through wearable technologies for older adults: smart wearables acceptance model. Appl Ergon.

[14] Nunes GS, Arruda Filho EJM (2018). Consumer behavior regarding wearable technologies: Google Glass. Innov Manag Rev.

[15] Dehghani M, Kim KJ (2019). The effects of design, size, and uniqueness of smartwatches: perspectives from current versus potential users. Behav Inform Technol.

[16] Heid M (2019). Are airpods and other bluetooth headphones safe?. Elemental.

[17] Cocosila M, Turel O, Archer N (2007). Perceived health risks of 3G cell phones: do users care?. Commun ACM.

[18] Duffy C (2020). Why conspiracy theorists think 5G is bad for your health and why experts say not to worry. CNN Business.

[19] Barua A, Al Alamin MA, Hossain M (2022). Security and privacy threats for bluetooth low energy in IoT and wearable devices: a comprehensive survey. IEEE Open J Commun Soc.

[20] Mathavan B, Vafaei-Zadeh A, Hanifah H (2024). Understanding the purchase intention of fitness wearables: using value-based adoption model. Asia-Pac J Bus Adm.

[21] Homburg C, Schwemmle M, Kuehnl C (2015). New product design: concept, measurement, and consequences. J Mark.

[22] Ajzen I (1991). The theory of planned behavior. Organ Behav Hum Decis Process.

[23] Luo X, Li H, Zhang J (2010). Examining multi-dimensional trust and multi-faceted risk in initial acceptance of emerging technologies: an empirical study of mobile banking services. Decis Support Syst.

[24] Hair JF, Ringle CM, Sarstedt M (2011). PLS-SEM: Indeed a Silver Bullet. J Mark Theory Pract.

[25] Jacoby J, Kaplan LB (1972). The components of perceived risk. ACR special volumes.

[26] Featherman MS, Pavlou PA (2003). Predicting e-services adoption: a perceived risk facets perspective. Int J Hum Comput Stud.

[27] Chin WW, Marcolin BL, Newsted PR (2003). A partial least squares latent variable modeling approach for measuring interaction effects: results from a Monte Carlo simulation study and an Electronic-Mail Emotion/Adoption study. Inf Syst J.

[28] Hair JF, Hult GTM, Ringle C (2016). A Primer on Partial Least Squares Structural Equation Modeling (PLS-SEM).

