# Supplementary material for: Perceived Risk and Fashion on the Intention to Adopt Wireless Earbuds in the United States Using a Partial Least Squares-Structural Equation Modeling Approach: Empirical Study
Source: JMIR Form Res. 2025 Jun 19;9:e56887. doi: 10.2196/56887 (PMC12199847; doi:10.2196/56887)
Supplement: Multimedia Appendix 1 [file formative-v9-e56887-s001.doc]

# **Appendix: Survey Items**

Seven-point Likert scale: Ranging from 1 (“strongly disagree”) to 7 (“strongly agree”)

*Perceived Health Risk (Adapted from Featehrman and Pavlou, 2003)*

PHR1. I believe wearing wireless earbuds is completely safe.

PHR2. I think wearing wireless earbuds could negatively affect my health.

PHR3. I think the more I use wireless earbuds, the more they would expose me to health risks.

PHR4. I think wearing wireless earbuds could increase the risk of cancer due to their wireless radiation.

*Perceived Privacy Risk (Adapted from Herz & Rauschnabel, 2019)*

PPR1. I think by using wireless earbuds, manufacturers could gather too much personal information about me.

PPR2. I think wireless earbuds could gather too much personal information about me.

PPR3. I think wireless earbuds could be easily hacked.

PPR4. I think hackers could target wireless earbuds to eventually hack smartphones connected to them.

PPR5. I think my privacy would be safe using wireless earbuds. *(Removed to improve reliability and validity)*

*Perceived Fashionability (Adapted from Herz & Rauschnabel, 2019)*

PF1. I think I would look good wearing wireless earbuds.

PF2. I think wireless earbuds are fashionable.

PF3. I think wearing wireless earbuds would make me look funny. *(Removed to improve reliability and validity)*

PF4. I don’t think wireless earbuds look appropriate to wear at work. *(Removed to improve reliability and validity)*

*Wearable Comfort (Adapted from Herz & Rauschnabel, 2019)*

WC1. I think wearing wireless earbuds would be comfortable.

WC2. I think wireless earbuds are light enough to wear them.

WC3. I think wireless earbuds would fit into my ears comfortably.

WC4. I don’t think it would be comfortable wearing wireless earbuds for a long period of time. *(Removed to improve reliability and validity)*

*Behavioral Intention to Purchase (Adapted from Herz & Rauschnabel, 2019)*

BITP1. I would (re)purchase wireless earbuds (repurchase additional pair for different occasions or upgrading to next model).

BITP2. I plan on (re)purchasing wireless earbuds.

BITP3. (Re)Purchasing wireless earbuds is a good idea.

*Behavioral Intention to Use (Adapted from Venkatesh et al., 2012)*

BITU1. I intend to continue using wireless earbuds in the future.

BITU2. I will use wireless earbuds instead of alternatives for my daily activities.

BITU3. I plan to continue to use wireless earbuds frequently.

*Controls*

OWN. Do you own wireless earbuds?

OWND. How long have you used wireless earbuds? (Answer by number of months)

APHR. Are you aware of the potential health risks associated with wireless earbuds?

AGE. How old are you?
